# RUNX1 inhibits the antiviral immune response against influenza A virus through attenuating type I interferon signaling

**DOI:** 10.1186/s12985-022-01764-8

**Published:** 2022-03-05

**Authors:** Yixiang Hu, Qi Pan, Kun Zhou, Yuehuan Ling, Hao Wang, Yan Li

**Affiliations:** 1 Department of Veterinary Medicine and Institute of Preventive Veterinary Sciences, Zhejiang University College of Animal Sciences, Hangzhou, 310058 Zhejiang China; 2 Zhejiang Provincial Key Laboratory of Preventive Veterinary Medicine, Hangzhou, 310058 Zhejiang China; 3 Hainan Institute, Zhejiang University, Sanya, 572025 Hainan China

**Keywords:** Influenza A virus, RUNX1, IFN, IRF3, STAT1, A549

## Abstract

**Background:**

Influenza A viruses (IAVs) are zoonotic, segmented negative-stranded RNA viruses. The rapid mutation of IAVs results in host immune response escape and antiviral drug and vaccine resistance. RUNX1 is a transcription factor that not only plays essential roles in hematopoiesis, but also functions as a regulator in inflammation. However, its role in the innate immunity to IAV infection has not been well studied.

**Methods:**

To investigate the effects of RUNX1 on IAV infection and explore the mechanisms that RUNX1 uses during IAV infection. We infected the human alveolar epithelial cell line (A549) with influenza virus A/Puerto Rico/8/34 (H1N1) (PR8) and examined RUNX1 expression by Western blot and qRT-PCR. We also knocked down or overexpressed RUNX1 in A549 cells, then evaluated viral replication by Western blot, qRT-PCR, and viral titration.

**Results:**

We found RUNX1 expression is induced by IAV H1N1 PR8 infection, but not by poly(I:C) treatment, in the human alveolar epithelial cell line A549. Knockdown of RUNX1 significantly inhibited IAV infection. Conversely, overexpression of RUNX1 efficiently promoted production of progeny viruses. Additionally, RUNX1 knockdown increased IFN-β and ISGs production while RUNX1 overexpression compromised IFN-β and ISGs production upon PR8 infection in A549 cells. We further showed that RUNX1 may attenuate the interferon signaling transduction by hampering the expression of IRF3 and STAT1 during IAV infection.

**Conclusions:**

Taken together, we found RUNX1 attenuates type I interferon signaling to facilitate IAV infection in A549 cells.

## Introduction

The Influenza A viruses (IAVs) are enveloped viruses within the family *Orthomyxoviridae*. The genome of IAVs, which are composed of eight segmented negative-stranded RNA (PB2, PB1, PA, HA, NA, NP, M, and NS), encode at least 17 proteins [1]. IAVs are contagious pathogens that can infect humans and a variety of animals, such as swine, poultry, equine, canine, and bats. In humans, IAVs primarily infect and replicate in epithelial cells of respiratory tract, resulting in alveolar epithelial cell injury and causes mild to severe pneumonia. Occasionally, IAV infection is associated with secondary bacterial infections, which may lead to fatal outcomes [2, 3].

During viral infection, the innate immune system is the host’s first line of defense against virus invasion [4]. Once IAV invades, viral pathogen-associated molecular patterns (PAMPs), such as viral RNAs and intermediate RNAs, are quickly recognized by the host through pathogen recognition receptors (PRRs), which include Toll-like receptors (TLRs), retinoic acid-inducible gene I (RIG-I)-like receptors, and nucleotide-binding oligomerization domain-like receptors [5]. Recognition of PAMPs by PPRs leads to innate immune signaling activation and transcriptional activity increase by transcription factors, such as IFN regulatory factor 3 (IRF3), nuclear factor kappa B (NF-κB), activator protein 1(AP1), and the coactivator CREB binding protein (CBP) and/or p300 [4]. Among these transcription factors, IRF3 is one of the most essential ones for interferon β (IFN-β) production. Upon viral infection, phosphorylated IRF3 forms complex with co-activator CBP/p300, then the complex translocates to the nucleus and binds the regulatory domains of the IFN-β promoter to initiate transcription of IFN-β [6]. IFN-β, which belongs to type I IFNs, is one of the key antiviral cytokines produced by influenza A virus-infected epithelial cells. The antiviral activity of type I IFN is mediated by a set of interferon-stimulated genes (ISGs) [7, 8]. Binding of IFN-β to its receptor is the initial step in this signaling process, followed by activation of the JAK family and subsequent activation of STAT1 protein that finally induces the production of ISGs which target different steps of the IAV life cycle [4, 9, 10].

The RUNX family consists of RUNX1, RUNX2, and RUNX3. In humans, RUNX1 is identified as one of the genes most frequently altered by chromosome translocation and point mutations in acute myelogenous leukemia (AML) [11, 12], so RUNX1 is also known as AML1. As a transcription factor, Runx1 has critical roles in embryonic and cell development, hematopoiesis, angiogenesis, tumorigenesis [13, 14]. Also, RUNX1 functions as a regulator in the inflammatory signaling. It suppresses LPS-induced inflammatory response in pulmonary diseases by inhibiting the NF-κB signaling pathway [15]. It also attenuates NF-κB signaling in myeloid cells through interaction with IκB kinase complex in the cytoplasm, so the AML1 mutant related leukemia cell exhibits distinctly activated NF-κB signaling [16]. However, it interacts with the NF-κB subunit p50 to enhance NF-κB mediated inflammatory signaling in macrophages upon LPS stimulation [17]. So the roles for RUNX1 in the inflammatory signaling are distinct in different situations. Beyond that, RUNX1 also plays an important role in HIV-1, SARS-CoV, Epstein-Barr virus, and VSV infection [18–26].

Like many other viruses, IAVs have evolved strategies to exploit and hijack the cellular machinery to escape innate immune response, especially to circumvent the type I IFN system [27]. Therefore, identifying and targeting the key inducible cellular factors that modulate IAV replication and pathogenesis would provide a potential solution to develop efficient antiviral drugs or vaccines [28–30].To identify cellular genes required in the IAV infectious cycle, several groups performed genome-wide screenings in different cells with different IAVs. Brass et al. conducted RNAi screens in osteosarcoma cells (U2OS) infected with influenza A/Puerto Rico/8/34 (H1N1) (PR8), and Karlas et al. performed RNAi screens in a human alveolar epithelial cell line (A549) with influenza A/WSN/33(H1N1) and the pandemic A/Hamburg/04/2009(H1N1) virus infection, and both of the groups identified RUNX1 as one of the targets [31, 32], which indicated that RUNX1 might promote IAV infection. [33]. Gaur et al. identified the interaction of NA protein of H1N1 PR8 virus with RUNX1 in A549 cells, and the knockdown of RUNX1 using siRNA resulted in decreased IFN-β expression in human myeloid leukemia cell line U937 [34]. However, the role and mechanism of RUNX1 in IAV H1N1 PR8 virus infection in A549 cells is not further studied.

Here, we investigated the effects and mechanisms of RUNX1 on IAV PR8 infection by both gain- and loss-of-function studies. We found that RUNX1 is induced by IAV PR8 infection in the A549 cells. The knockdown of RUNX1 significantly inhibited IAV infection, while overexpression of RUNX1 promoted IAV infection. Moreover, induction of RUNX1 inhibited the expression of IFN signaling related proteins IRF3 and STAT1, and influenced the production of IFN-β and ISGs. Our results demonstrated that RUNX1, acting as a negative regulator of the IFN signaling pathway, is capable of attenuating antiviral defenses and facilitating IAV infection.

## Materials and methods

### Cell culture and virus

Human alveolar adenocarcinoma epithelial (A549) cells, human embryonic kidney (HEK 293T) cells, and Madin-Darby canine kidney (MDCK) cells were used in this study. A549 cells were maintained in the RPMI 1640 (Thermo Fisher, Waltham, MA, USA) plus 10% fetal bovine serum (FBS) (ExCell Biology, shanghai, China). 293T cells were cultured in the Dulbecco’s modified Eagle’s medium (DMEM) (Thermo Fisher, Waltham, MA, USA) supplemented with 10% FBS and 100 U/mL penicillin and 0.1 mg/mL streptomycin (Thermo Fisher, Waltham, MA, USA). The MDCK cells were grown in minimum essential medium (MEM) (Thermo Fisher, Waltham, MA, USA) containing 10% FBS, 1 mM sodium pyruvate (Thermo Fisher, Waltham, MA, USA), 100U/mL penicillin, and 0.1 mg/mL streptomycin.

The influenza virus A/Puerto Rico/8/34 (H1N1) (PR8), A/Zhejiang/163/2020 (H3N2) (ZJ163), A/swine/Jiangsu/C1/2008 (H9N2) (JSC1) and A/California/04/2009(H1N1) (CA04) was propagated in 10-day-old chicken embryos, and viral titers were determined by calculating the 50% tissue culture infectious dose (TCID_50_) per milliliter using the Reed-Muench method in MDCK cells.

### Antibodies

Mouse anti-RUNX1(A-2) (sc-365644) antibodies were purchased from Santa Cruz, Dallas, TX, USA; rabbit anti-IRF3 (11312-AP) and rabbit anti-STAT1 (10144–2-AP) were purchased from Proteintech, Rosemont, IL, USA; rabbit anti-P-IRF3 (Ser396) (4D4G) antibodies and rabbit anti-P-STAT1 (Tyr701) (D4A7) antibodies were purchased from Cell Signaling, Danvers, MA, USA; mouse anti-GAPDH antibody (AF0006) was purchased from Beyotime, Shanghai, China; mouse mAbs to viral proteins NP and M1 of IAV were obtained from Dr. Jiyong Zhou [35].

### ***Viral infection and TCID***_***50***_*** assay***

When the MDCK cells grew to ~ 95% confluent monolayers, the cell culture medium was withdrawn and the cells were washed twice with phosphate-buffered saline (PBS), and then viruses in viral growth medium were inoculated in the cells. After adsorption at 37 ℃ for 1 h, the inoculum was removed and replaced with a viral growth medium. The viral growth medium was a serum-free medium supplemented with 2% BSA V and 2 μg/mL of tosylsulfonyl phenylalanyl chloromethyl ketone (TPCK)-trypsin (Sigma, St. Louis, MO, USA).

To measure the infectious titer of virus in cell culture supernatants, MDCK cells were plated in 96-well plates and grow to 90–95% confluence overnight. The collected viral supernatants were immediately serially diluted in a serum-free medium. MDCK cells were then infected as described above. IAV-induced cytopathic effect (CPE) was monitored for 24–96 h. TCID_50_ was then calculated by the Reed-Muench formula.

### Vector construction and transfections

The wild-type *RUNX1* gene was obtained from the THP-1 human monocytic leukemia cell line by RT-PCR and subsequently subcloned into pCMV expression vector via *Xba*I and *BamH*I sites. Corresponding primers used were as follows: pCMV-RUNX1 CDS forward, 5′-ATG GCT TCA GAC AGC ATA TT-3′, reverse, 5′-TCA GTA GGG CCT CCA CAC GG-3′. Recombinant plasmids were confirmed by DNA sequencing and plasmids were prepared by using OMEGA Endo-Free Plasmid Mini Kit I (OMEGA Bio-Tek, Norcross, GA, USA). Transfection of plasmids to 80 ~ 90% confluent cell monolayers was performed using the Lipofectamine 2000 transfection reagent (Thermo Fisher, Waltham, MA, USA) according to the manufacturer’s instructions. Polyinosinic-polycytidylic acid (poly (I:C)) (Tocris Bioscience, Bristol, UK) was introduced to the cells with or without Lipofectamine 2000 transfection reagent.

### Generation of stable cell lines with RUNX1 knockdown

To generate the stable cell lines that have RUNX1 knocked down, A549 cells were transduced with pseudotyped lentiviruses which express RUNX1-shRNA or Luci-shRNA (Luci is short for luciferase) as control. We used the third generation of lentivirus packaging system that contains three packaging plasmids (pGag/Pol, pRev, and pVSV-G) and one expression plasmid (shRNA-pCD513B-1 plasmid containing Puro and GFP hU6 promoter) to produce the lentiviruses we needed. The sequences for RUNX1-shRNA and Luci-shRNA were designed by using Invitrogen online shRNA design software and synthesized and subsequently subcloned into pCD513B-1 expression vectors via *Spe*I and *BamH*I. The sequences are as follows: RUNX1-shRNA: 5′-GAA CCA GGT TGC AAG ATT TAA-3′; Luci-shRNA: 5′-CGT ACG CGG AAT ACT TCG A-3′; the loop: 5′-CTC GAG-3′. Plasmids for RUNX1shRNA or Luci-shRNA expression combined with three packaging plasmids were mixed with Lipofectamine 2000 (Thermo Fisher, Waltham, MA, USA) and transfected into HEK293T cells according to the manufacturer’s protocol. The transfection medium was replaced with advanced DMEM supplemented with 2% FBS, 0.01 mM L-a-phosphatidylcholine, 0.01 mM cholesterol (Sigma, St. Louis, MO, USA), 4.0 mM L-glutamine, and 1:1000 diluted chemically defined lipid (Thermo Fisher, Waltham, MA, USA) at 16 h post-transfection. The cell-free supernatant that contains lentiviruses were collected at 48 h after transfection and lentiviral titers were determined by TCID_50_ assay in HEK293T cells. To generate A549 cells that stably express RUNX1-shRNA or Luci-shRNA, A549 cells in six-well plates were inoculated with lentivirus at a multiplicity of infection (MOI) of and 4 mL RPMI 1640 fresh medium plus 10% FBS. After incubation at 37 ℃ for 12 h, the inoculum was removed and fresh medium was added, and the cells were incubated at 37 ℃ for another 48 h. The lentivirus-infected A549 cells were selected by supplementation of 2 g/mL puromycin (Sigma, St. Louis, MO, USA) in the medium to generate the shRUNX1 or shControl A549 stable cell line. Since the shRNA expression plasmid contains a GFP reporter, the green fluorescence was detected under a fluorescence microscope (Olympus, Tokyo, Japan) to determine the transduction efficiency. Western blot and qRT-PCR were used to examine the level of *RUNX1* expression. After *RUNX1* knockdown efficiency was determined, the cell lines were used to perform further experiments.

### Reverse transcription and quantitative real-time PCR

A two-step real-time quantitative RT-PCR was used to examine specific mRNA levels. For qRT-PCR analysis, total RNAs were prepared by RNA preparation kit (TransGen, Beijing, China). Reverse transcription was carried out with HiScript Q Select RT SuperMix for qPCR (+gDNA wiper) (Vazyme, Nanjing, China). The primers of RT-PCR were designed using PrimerQuest Tool. The sequences of primers for *β-Actin, RUNX1, IFNB1, ISG15, MxA, TRAF3, RIG-I, MAVS, TBK1, IRF3, STAT1, PR8-M,* and *PR8-NP* are listed in Table 1. Each gene was amplified in triplicate and mean threshold (Ct) values were calculated. The housekeeping gene *β-Actin* was used for normalization in gene expression analysis. Relative fold changes in gene expression among groups were determined using the 2^−ΔΔCt^ method.

**Table 1:** Primers used for qPCR

Target gene	Direction	Sequence (5′–3′)
*β-Actin*	Forward	ATCTGGCACCACACCTTCTACAATGAGCTGCG
*β-Actin*	Reverse	CGTCATACTCCTGCTTGCTGATCCACATCTG
*RUNX1*	Forward	CTTTCAAGGTGGTGGCCCTA
*RUNX1*	Reverse	CTTGCGGTGGGTTTGTGAAG
*IFNB1*	Forward	TTGTTGAGAACCTCCTGGCT
*IFNB1*	Reverse	TGACTATGGTCCAGGCACAG
*ISG15*	Forward	CGCAGATCACCCAGAAGATCG
*ISG15*	Reverse	TTCGTCGCATTTGTCCACCA
*MxA*	Forward	GTTTCCGAAGTGGACATCGCA
*MxA*	Reverse	GAAGGGAACTCCTGACAGT
*TRAF3*	Forward	GCGTGTCAAGAGAGCATCGTT
*TRAF3*	Reverse	GCAGATGTCCCAGCATTAACT
*RIG-I*	Forward	ACGCAGCCTGCAAGCCTTCC
*RIG-I*	Reverse	TGTGGCAGCCTCCATTGGGC
*MAVS*	Forward	CAGGCCGAGCCTATCATCTG
*MAVS*	Reverse	GGGCTTTGAGCTAGTTGGCA
*TBK1*	Forward	GGCGGCTAGAAGAGGCTTTG
*TBK1*	Reverse	CTCCGTCAGCTCGGTGTAG
*IRF3*	Forward	GCAGGAGGATTTCGGAATCTTC
*IRF3*	Reverse	GGAAATTCCTCTTCCAGGTTGG
*STAT1*	Forward	TGGCCCTAAAGGAACTGGAT
*STAT1*	Reverse	CACTATCCGAGACACCTCGTC
*PR8-NP*	Forward	TGCCTGTGTGTATGGACCTG
*PR8-NP*	Reverse	TTCAAATGCGGCAGAATGGC
*PR8-M*	Forward	CCAGCATCGGTCTCATAGGC
*PR8-M*	Reverse	TCGATCCAGCCATTTGCTCC

### Western blot analysis

Cells were collected and washed with PBS, and then lysed with RIPA lysis buffer (Beyotime, Shanghai, China) containing a protease inhibitor cocktail (Roche, Basel, Switzerland). The cell lysates were centrifuged at 12,000* g* for 10 min at 4 ℃ and the supernatant was collected. Equal amounts of protein samples were subjected to SDS–polyacrylamide gel electrophoresis and transferred to PVDF membrane (Millipore, Darmstadt, Germany). The membrane was probed with various primary antibodies as indicated and detected using the ECL system with alkaline phosphatase-conjugated secondary antibodies according to the manufacturer’s protocol.

### Statistical analysis

All data were expressed as mean x ± standard deviation (SD) from at least three independent experiments. The statistical analyses were performed with a two-tailed Student’s *t*-test and a two-way ANOVA test. *p* < 0.05 was considered statistically significant. Differences between groups were considered significant if *p < 0.05, highly significant if **p < 0.01 and extremely significant if ***p < 0.001.

## Results

### RUNX1 is induced by IAV PR8 infection in A549 cells

**Fig. 1:**
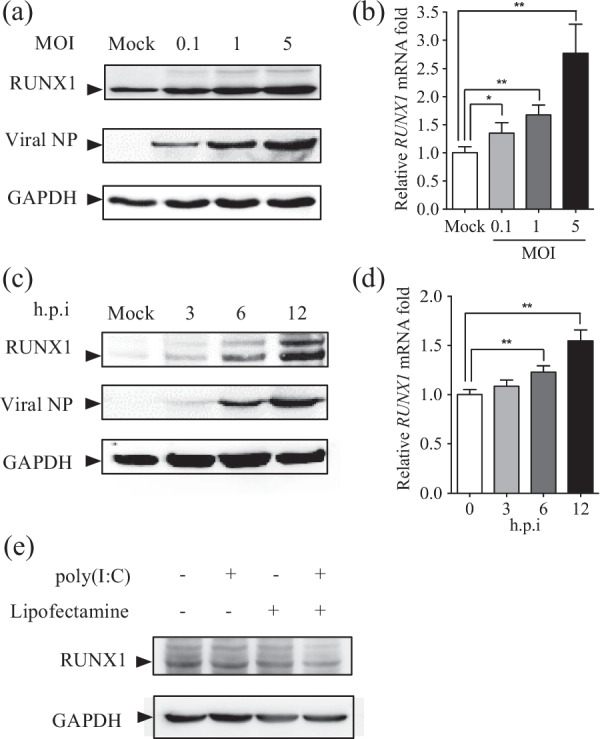
Influenza virus A/Puerto Rico/8/34 (H1N1) (PR8) infection induced RUNX1 expression. **a** A549 cells were infected with PR8 at indicated MOIs and harvested at 12 h.p.i., protein levels of RUNX1, vial NP, and GAPDH were analyzed by Western blot. **b** A549 cells were infected with PR8 at indicated MOIs and harvested at 12 h.p.i., and RUNX1 and GAPDH mRNA levels were analyzed by qRT-PCR. **c** A549 cells were infected with IAV at an MOI of 1. Cells were harvested at 3, 6, and 12 h.p.i. and analyzed by Western blot with anti-RUNX1, anti-NP, and anti-GAPDH antibodies. **d** A549 cells were infected with PR8 at an MOI of 1. Cells were harvested at 3, 6, and 12 h.p.i. and RUNX1 and GAPDH mRNA levels were analyzed by qRT-PCR. **e** A549 cells were treated with 1 μg/ml poly(I:C) with (+) or without (−) transfection reagent lipofectamine 2000 for 12 h and collected for Western blot analysis of RUNX1. Data are mean ± SD of three independent experiments. Significance is by two-way ANOVA test; *p < 0.05, **p < 0.01

We first examined whether IAVs could induce RUNX1 expression. A549 cells were infected with PR8 at MOI of 0.1, 1, and 5, and the expression of *RUNX1* was detected by Western blot and qRT-PCR at 12 h post-infection (h.p.i.). Increases of RUNX1 protein and mRNA were observed in PR8-infected samples, and the elevation was dose-dependent (Fig. 1a, b). We also examined RUNX1 expression in A549 cells at different time points after PR8 infection. A549 cells were infected with PR8 at an MOI of 1 and were collected at 3, 6, and 12 h.p.i.. The results from the Western blot and qRT-PCR experiments showed that RUNX1 protein synthesis gradually increased and the mRNA level significantly increased about 1.5-fold at 12 h.p.i. (*p* < 0.01) (Fig. 1c, d). RUNX3, a developmental regulator and tumor suppressor that belongs to RUNX family, was induced by IAV H1N1 and H3N2, influenza viral RNA, a synthetic analog of viral double-stranded RNA (dsRNA) polyinosinic-polycytidylic acid (poly(I:C)) in the normal human bronchial epithelial cell line BEAS-2B [36]. But RUNX1 was not induced by poly(I:C) in A549 cells (Fig. 1e). Taken together, these data demonstrated that PR8 infection induced RUNX1 expression.

### RUNX1 knockdown reduced virus replication

**Fig. 2:**
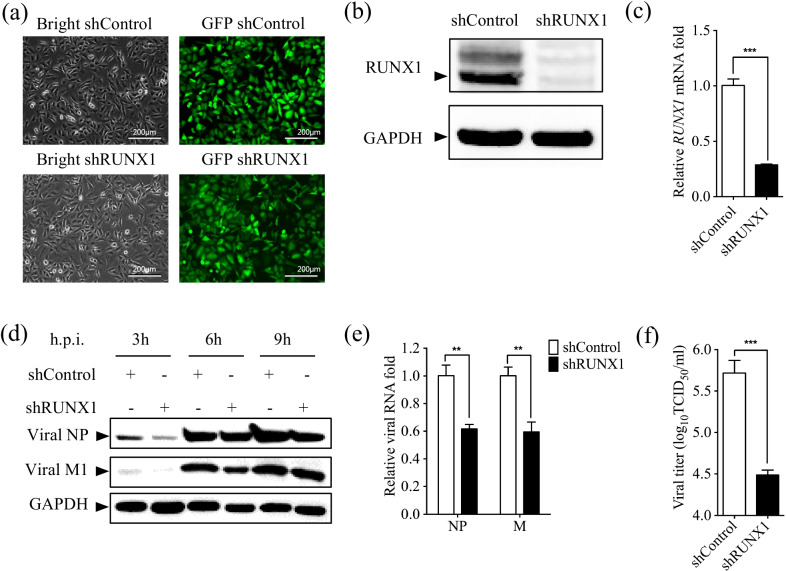
Knockdown of RUNX1 inhibits PR8 replication. **a** Stable transfection of A549 cells with RUNX1 shRNA. Bright-field microscopy of and fluorescent microscopy of cells transfected with shControl and shRUNX1 were applied to check the cell growth and GFP expression. **b** The RUNX1 protein was assessed by Western blot. (**c**) The mRNA of RUNX1 was assessed by qRT-PCR. **d** The shRUNX1 cells and shControl cells were infected with PR8 at an MOI of 1 and collected at 3, 6, and 9 h.p.i.. The viral NP and M1 protein were assessed by Western blot. **e** The shRUNX1 cells and shControl cells were infected with PR8 at an MOI of 1 and collected at 8 h.p.i.. The RNA of viral NP and M was assessed by qRT-PCR. **f** The shRUNX1 cells and shControl cells were infected with PR8 (MOI = 0.01) and the culture supernatants were collected at 24 h.p.i. for viral titration by TCID_50_ assay. All images were captured at × 200 magnification, scale bars = 100 μm. Data are mean ± SD of three independent experiments. Significance is by unpaired T-test; **p < 0.01. ***p < 0.001

The aforementioned data showed that H1N1 PR8 viruses infection induced RUNX1 expression in A549 cells, so we hypothesized that RUNX1 may play a role in influenza virus infection. To test this hypothesis, RUNX1 was knocked down in A549 cells by transduction of lentiviruses (Fig. 2a). Results from Western blot and qRT-PCR showed that the level of RUNX1 protein and mRNA was significantly reduced (Fig. 2b, c). Next, we examined the progeny virus production in the shRUNX1 and shControl cells infected with PR8 at an MOI of 1, and harvested the cells at 0, 3, 6, and 9 h.p.i. and examined viral NP and M1 protein by Western blot and qRT-PCR. In comparison with the control cells, knockdown of RUNX1 led to a significant reduction of viral M1 protein expression and slight reduction of viral NP protein expression (Fig. 2d), and the RNA of NP and M decreased to 60% correspondingly in shRUNX1 cells at 8 h.p.i (Fig. 2e). Also, we examined the infectious progeny virus titers in the supernatant collected from shRUNX1 and shControl cells infected with PR8 at an MOI of 0.01. The results showed that virus titer in shRUNX1 cells supernatant was about 17-fold lower than that in shControl cells after 24 h infection (Fig. 2f). These results demonstrated that knockdown of RUNX1 had an inhibitory effect on the PR8 virus life cycle.

### RUNX1 overexpression facilitates IAV replication

**Fig. 3:**
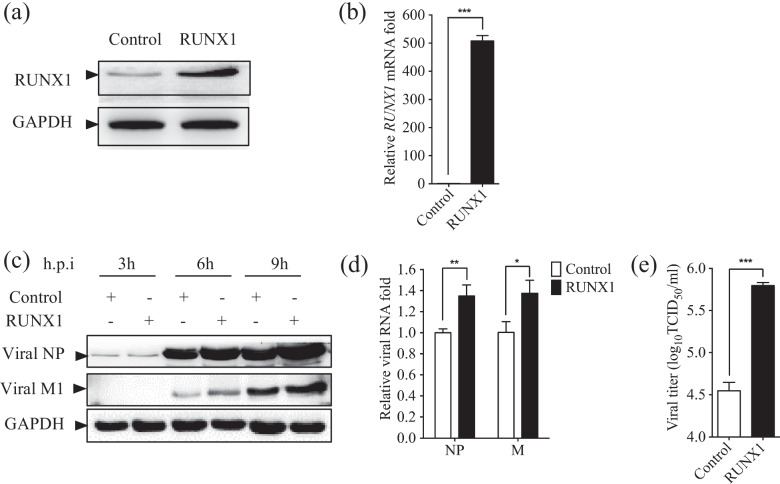
Overexpression of RUNX1 facilitates PR8 replication. A549 cells were transfected with vector plasmid pCMV-GFP or pCMV-RUNX1. **a** RUNX1 overexpression efficiency was detected by Western blot. **b** RUNX1 overexpression efficiency was detected by qRT-PCR. **c** These cells were infected with PR8 (MOI = 1) and collected at 3, 6, and 9 h.p.i. The viral NP and M1 protein were assessed by Western blot. **d** These cells were infected with PR8 (MOI = 1) and collected at 8 h.p.i. The viral NP and M RNA was assessed by qRT-PCR. **e** Culture supernatants were collected for viral titration by TCID_50_ assay at 24 h.p.i. Data are mean ± SD of three independent experiments. Significance is by unpaired T-test; *p < 0.05; **p < 0.01; ***p < 0.001

To validate the results that knockdown of RUNX1 impaired IAV PR8 progeny virus production, we performed similar experiments using the A549 cells which transiently overexpressed RUNX1 and the A549 cells which were transfected with an empty vector to serve as the control. Overexpression of RUNX1 was confirmed by Western blot and qRT-PCR at 24 h.p.i. (Fig. 3a, b). To examine the effect of RUNX1 overexpression on IAV replication, we applied Western blot and qRT-PCR to detected viral NP and M in the infected cells. In consistent with the observations we saw in the shRUNX1 cells, more viral NP and M production was found in the cells with RUNX1 overexpression (Fig. 3c, d). To further confirm the impact of RUNX1 on IAV replication, we collected the supernatant and titrated the viruses by TCID_50_ assay. The results showed that viral titer of PR8 in RUNX1-overexpressing cells was also increased ~ 18 fold in comparison with that in control cells (Fig. 3e). Collectively, these results demonstrated that RUNX1 enhanced PR8 infection and progeny virus production in host cells.

### RUNX1 negatively regulates IAVs-induced IFN-β and ISGs expression

**Fig. 4:**
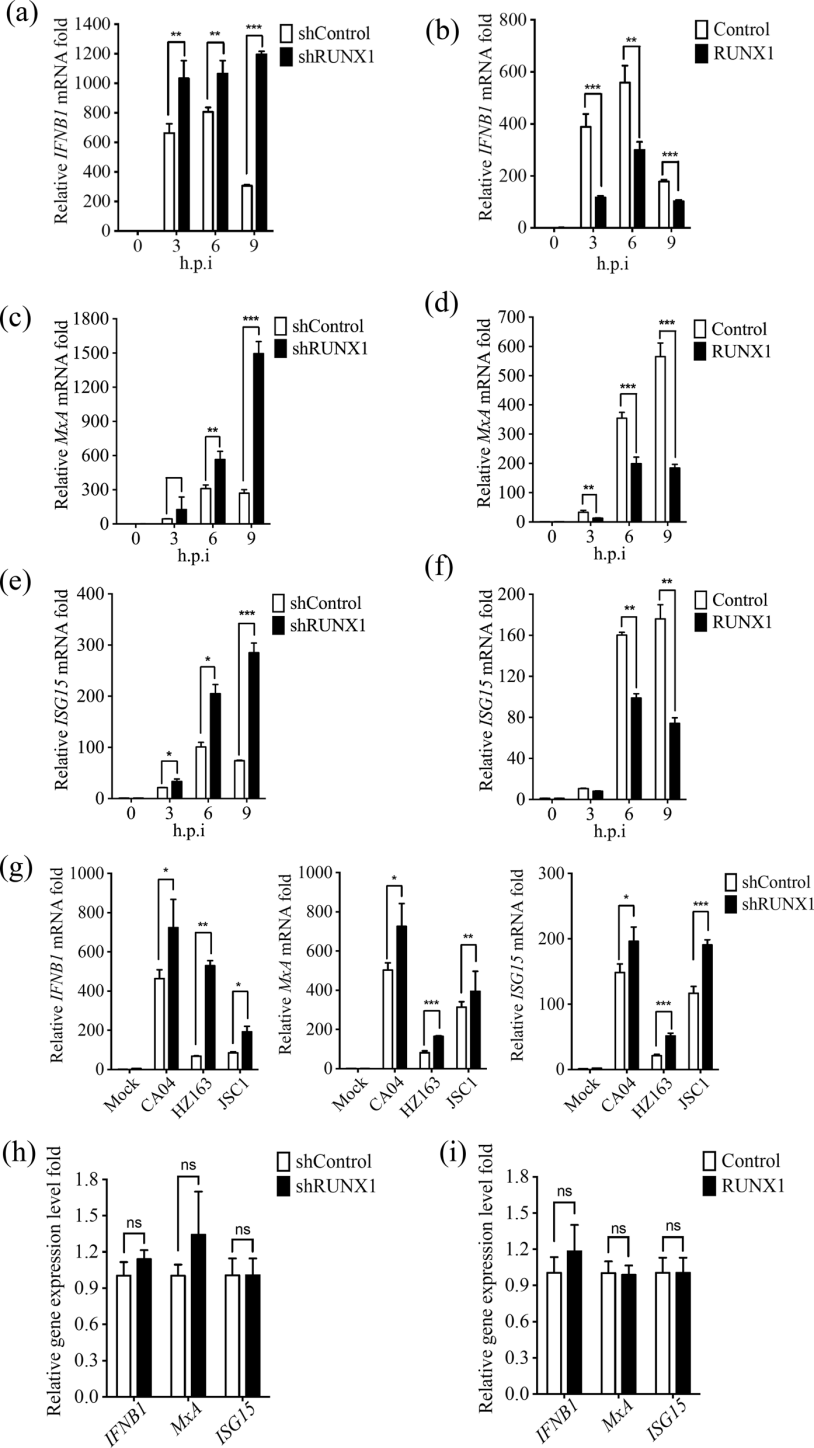
RUNX1 attenuates PR8-induced IFN-β and ISG expression. **a**, **c**, **e** The shRUNX1 cells and shControl cells were infected with PR8 (MOI = 5) and harvested at 3, 6, and 9 h.p.i. The mRNA level of *IFNB1*, *MxA* and *ISG15* was assessed by qRT-PCR. **b**, **d**, **f** The A549 cells which were transfected with empty plasmid pCMV-GFP or pCMV-RUNX1. After 24 h transfection, the cells were infected with PR8 (MOI = 5) and harvested at 3, 6, and 9 h.p.i. The mRNA level of *IFNB1*, *MxA,* and *ISG15* was assessed by qRT-PCR. (**g**) The shRUNX1 cells and shControl cells were infected with influenza A/Zhejiang/163/2020 (H3N2) (ZJ163), A/swine/Jiangsu/C1/2008 (H9N2) (JSC1) and A/California/04/2009(H1N1) (CA04) (MOI = 1) and harvested at 9 h.p.i. The mRNA level of *IFNB1*, *MxA* and *ISG15* in the A549 cells was assessed by qRT-PCR. **h** The mRNA level of *IFNB1*, *MxA,* and *ISG15* in the shRUNX1 cells and shControl cells without viral infection was assessed by qRT-PCR. **i** The mRNA level of *IFNB1*, *MxA,* and *ISG15* in the A549 cells which were transfected with empty plasmid pCMV-GFP or pCMV-RUNX1 was assessed by qRT-PCR. Data are mean ± SD of three independent experiments. Significance is by unpaired T-test; *p < 0.05; **p < 0.01. ***p < 0.001

Our results have shown that RUNX1 regulates IAV PR8 infection. Interferons are the first line of defense against viral infection and IFN-β plays key roles in anti-IAVs infection [5, 37]. It is also demonstrated that RUNX1 negatively regulates innate immune responses during viral infection [26]. We next investigated the effects of RUNX1 on IFN-β expression upon IAV infection. We performed qRT-PCR to compare the mRNA levels of *IFNB1* between RUNX1-knockdown and control cells after PR8 infection. shRUNX1 A549 cells and shControl A549 cells were infected with PR8 at an MOI of 5, and then the cells were collected at 0, 3, 6, h.p.i. Compared with shControl cells, the *IFNB1* mRNA levels in the shRUNX1 cells significantly increased upon PR8 infection, and increased fold is ~ 4 at 9 h post PR8 infection (Fig. 4a). The results revealed that knockdown of RUNX1 in A549 cells augmented IAV-induced IFN-β expression. To further gain insight into the antiviral state, we also analyzed the mRNA levels of *MxA* and *ISG15,* which have been reported to be involved in IAV infection [38–41]. The results showed that the expression of *MxA* and *ISG15* increased 3 ~ 6 fold in shRUNX1 A549 cells as compared with that in control cells (Fig. 4c, e). We also validated the results by overexpression of RUNX1 in the A549 cells. Accordingly, cells transfected with RUNX1 expression plasmids or vector-only plasmids were infected with PR8 at an MOI of 5, and the cells were collected at 0, 3, 6, 9 h.p.i. for qRT-PCR analysis of *IFNB1*, *MxA* and *ISG15*. Similarly, overexpression of RUNX1 significantly reduced *IFNB1*, *MxA*, and *ISG15* mRNA levels upon PR8 infection (Fig. 4b, d, f). These data indicated that RUNX1 may negatively regulate IAV-induced IFN-β responses.

To further investigate whether RUNX1 negatively regulate IFN-β signaling upon IAV infection in A549 cells is not strain specific, we infected the A549 cells with influenza A/Zhejiang/163/2020 (H3N2) (ZJ163), A/swine/Jiangsu/C1/2008 (H9N2) (JSC1) and A/California/04/2009(H1N1) (CA04) at MOI of 1, and then examined the *IFNB1*, *MxA*, and *ISG15* expression at 9 h.p.i. We found that the expression of *IFNB1*, *MxA*, and *ISG15* increased in the RUNX1 knockdown cells by 2 ~ ninefold compared to the control cells (Fig. 4g).

Interferons and ISGs are very crucial in the anti-viral immunity. To test whether RUNX1 could directly regulate IFN-β signaling, we analyzed the expression of *IFNB1*, *MxA* and *ISG15* in the A549 cells without viral infection. As shown in Fig. 4h, i, the expressions of *IFNB1*, *MxA* and *ISG15* did not altered no matter RUNX1 was knocked down or overexpressed. It suggested that RUNX1 does not directly regulate IFN-β and ISGs expression.

### RUNX1 suppresses IRF3- and STAT1-mediated signaling pathways

**Fig. 5:**
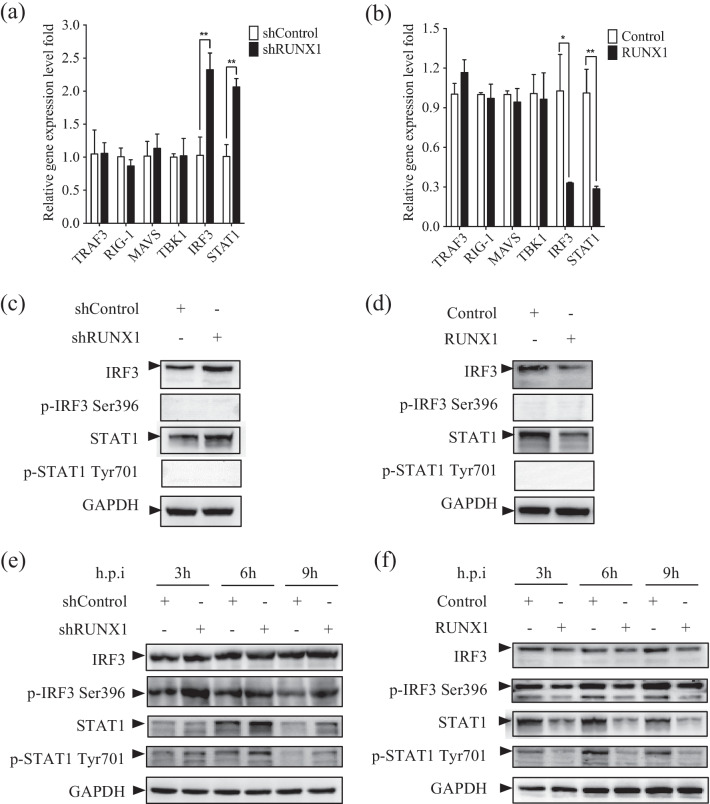
RUNX1 attenuated IRF3 and STAT1 signaling. **a** The mRNA level of *TRAF3*, *RIG-I*, *MAVS*, *TBK1*, *IRF3,* and *STAT1* in the shRUNX1 and shControl cells were assessed by qRT-PCR. **b** The mRNA of these genes in A549 cells that were transfected with empty plasmid pCMV-GFP or pCMV-RUNX1 was assessed by qRT-PCR. **c**, **d** The protein and the phosphorylation level of IRF3 and STAT1 of these cells were assessed by Western blot. **e**, **f** These cells were infected with PR8 (MOI = 5) and collected at 3, 6, and 9 h.p.i. The protein and the phosphorylation levels of IRF3 and STAT1 in these cells were assessed by Western blot. Data are mean ± SD of three independent experiments. Significance is by unpaired T-test; *p < 0.05; **p < 0.01

In view of the incapability of RUNX1 in direct regulation of IFN-β and ISGs expression, we further elucidate the molecular mechanism that how RUNX1 regulates IFN-β production, we first analyzed the expression of type I IFN signaling-related genes (*TRAF3, RIG-I, MAVS, TBK1, IRF3, and STAT1)* in A549 cells by qRT-PCR [6, 27, 42, 44]. Results from qRT-PCR assay showed that the mRNA levels of *IRF3* and *STAT1*, whose phosphorylation is important in IFN-β and ISGs production[5, 9, 42, 43], were higher in the A549 cells with RUNX1 knockdown (Fig. 5a), while their level decreased in the A549 cells with RUNX1 overexpression (Fig. 5b). Consistently, knockdown of RUNX1 increased IRF3 and STAT1 protein levels (Fig. 5c) and overexpression of RUNX1 decreased IRF3 and STAT1 protein levels (Fig. 5d). Phosphorylation of IRF3 and STAT1 is essential in the activation of IFN-β and ISGs expression [9, 43, 45, 46]. We also tested the role of RUNX1 on phosphorylation of IRF3 and STAT1 in non-infected A549 cells to exclude the possibility that RUNX1 would impair the phosphorylation of IRF3 and STAT1 in these cells. We did not detect phosphorylation of IRF3 and STAT1 in A549 cells of RUNX1 knockdown or overexpression without virus infection (Fig. 5c, d).

To address whether RUNX1 could indirectly modify phosphorylation of IRF3 and STAT1 upon viral infection, we infected the cells with PR8 at an MOI of 5, collected the cells at 3, 6, and 9 h.p.i. and assessed the phosphorylation of IRF3 and STAT1. As shown in Fig. 5e, phosphorylation of IRF3 and STAT1 was enhanced when RUNX1 was knocked down, while phosphorylation of IRF3 and STAT1 were markedly decreased in the cells with RUNX1 overexpression compared with the control cells at 3, 6, and 9 h post PR8 infection (Fig. 5f). These data demonstrated that RUNX1 inhibited IRF3 and STAT1 signaling.

## Discussion

Due to the relatively small genome and a limited number of protein-coding genes, influenza A viruses have to depend on the host to replicate and complete its life cycle [47]. Several studies have suggested that influenza viruses have evolved ways to hijack host factors and to alter host cell metabolism to facilitate viral replication [1, 48]. For antiviral drug development, it is highly necessary to identify these cellular factors which modulate IAV replication and pathogenesis. In the present study, we identified RUNX1 is an important transcription factor that facilitates replication of H1N1 PR8. It is interesting to know whether the effects of RUNX1 on IAV infection is widely applied by other influenza virus strains. We found that when the cells were infected with H1N1 CA04, H3N2 ZJ163 and H9N2 JSC1, the expression of the *IFNB1, MxA*, and *ISG15* increased in the RUNX1 knockdown cells. It indicated RUNX1 would facilitate H1N1 CA04, H3N2 ZJ163 and H9N2 JSC1 infection as well, because IFN-β and ISGs are crucial in the antiviral response. Several studies applied genome-wide screens to identify the cellular factor involved in influenza infection and replication with different cells and different influenza strains, and only two groups who used H1N1 identified RUNX1 is one of the hundreds of targets [31–33, 49–53]. Maybe because RUNX1 expression levels are variable in different cells. Here we found that RUNX1 expression is induced by IAV H1N1 PR8 infection in A549 cells. Gan et al. did not detect increased RUNX1 expression in BEAS-2B cells upon H1N1 PR8 infection [36]. The difference may be resulted from the different cells applied. A549 cells are the tumor cells, while BEAS-2B are the normal human bronchial epithelial cell. The intrinsic activities of genes in different cells may be distinct.

Enormous studies have indicated that transcription factor RUNX1 orchestrates many different aspects of biology, including basic cellular and developmental processes, stem cell biology, tumorigenesis, and immunity [54, 55]. Besides, RUNX1 also plays role in viral infection. RUNX1 regulates apoptosis during transmissible gastroenteritis virus infection [22]; Runx1 activates polyomavirus DNA replication by stimulating the binding of the viral-encoded replication initiator/helicase, large T antigen, to its replication origin [24]. RUNX1 also interacts with SARS-CoV accessory protein 3b interact RUNX1 to enhance transcription of macrophage inflammatory protein (MIP-1α) [20]. However, little is known about the innate immune mechanism regulated by RUNX1 during IAV infection. In this study, we found that RUNX1 expression can be induced with time and the dose-dependent manner by H1N1 PR8 infection in A549 cells. But we do not know exactly how IAV induced RUNX1 expression. Many studies suggest that RUNX1 functions as a key player in the replication of various viruses, such as polyomavirus [24], HIV [56], and alphaherpesvirus [57]. Gaur et al. found IAV NA protein interacts with RUNX1 [34]. Our previous study showed that type I and type II IFNs may regulate RUNX1 expression in the embryonic aorta [58]. Upon IAV infection, viral RNA could be rapidly recognized by the PRRs, and type I IFNs was rapidly induced in the cells (Fig. 4a); nevertheless, we did not detect increased RUNX1 expression in A549 cells when treated with poly(I:C), which is a strong type I IFN inducer. Our result was consistent with what Gan et al. observed in BEAS-2B. The results suggested that RUNX1 induction might rely on some steps in the IAV life cycle. The mechanisms are worth future studies.

Our study has also addressed that RUNX1 may facilitate IAV infection by modulating host innate immune systems. We observed that when RUNX1 was knocked down, IAV replication was impaired along with significantly increased IFN-β expression. While overexpression of RUNX1 led to the opposite consequences. These findings suggested that RUNX1 attenuated the IFN-β production in epithelial cells upon IAV infection. However Gaur et al. recently reported that decreased expression of IFN-β in IAV infected U937 cells where AML1 was knocked down using siRNA [34]. These contradictory observations might be due to the different type of cells used. Previous studies have shown that RUNX1 plays an opposite role in the regulation of inflammatory response in the epithelial cell and macrophages in the lung [15, 17, 59]. These results served as a reminder that RUNX1 may play a dual role in the different cells in the lungs from IAV infected patients. Our results also provided new insights into the role of RUNX1 in regulating immune function in pulmonary disease caused by IAV infection. Furthermore, we examined the effect of RUNX1 on IFN downstream effectors, such as ISG15 and MxA. Overexpression of RUNX1 significantly impaired IAV-induced ISGs expression and knockdown of RUNX1 weakened this inhibition. However, the expression of IFNB1, MxA and ISG15 did not changed in the non-infected A549 cells no matter we knocked down or overexpressed RUNX1. These results indicated that IAV-induced RUNX1 antagonized the innate immunity response by suppressing IAV-induced expression of IFN-β and ISGs, and RUNX1 could not directly regulate IFN-β and ISG expression.

To further explore the mechanism by which RUNX1 regulates the IFN signaling, we examined the effect of RUNX1 on the expression of IFN signaling pathway-related proteins (TRAF3, RIG-I, MAVS, TBK1, IRF3, STAT1). We observed that overexpression of RUNX1 inhibited IRF3 and STAT1 expression, while knockdown of RUNX1 increased IRF3 and STAT1 expression in A549 cells, no matter whether the A549 cells were infected by IAV or not. Correspondingly, the level of phosphorylated IRF3 and STAT1 changed in the same way in the cells upon IAV infection. Based on the level changed as reflected by Western blot, we supposed that the changed levels of IRF3 and STAT1 phosphorylation in RUNX1-knockdown or overexpression A549 cells during PR8 infection are mainly due to the changed IRF3 and STAT1 expression. Previous studies have reported that IAVs inhibited IRF3 and STAT1 signal transduction by utilizing a variety of host proteins, such as PGRN and A20, to block IFN signaling transduction [60, 61]. Maybe RUNX1 aid IAV infection by impairment of IFN-β and ISGs production through inhibition of STAT1 and IRF3. As a DNA-binding transcription factor that regulates genes expression, RUNX1 must gain access to its binding sites within a chromatin context, and then it recruits many other coactivators to promote gene expression [62]. In addition, RUNX1 can serve as transcription repressor by recruiting corepressors to target genes. For example, RUNX1 binds to corepressor SIN3A complex [63] and the Groucho/TLE repressor complex [64, 65]. Whether RUNX1 active or suppress gene expression, it depends on the cellular and promoter context. Our results in this study indicated that RUNX1 negatively regulates IRF3 and STAT1. To find out whether RUNX1 could directly regulate IRF3 and STAT1 expression, we searched the RUNX1 binding site in the DNA by CHIP-Atlas database (http://chip-atlas.org), and found that RUNX1 can bind many regions located in promoters and gene bodies of STAT1 (chr2:191013544–191014873 and 190979768–190981151) and IRF3 (chr19:49665448–49666448, 49664233–49665075, 49661796–49662665, and 49658977–49659365), as well as some regions upstream or downstream 10 K far away from gene bodies. It is worthwhile to study further by using ChIP-Seq to examine the histone modifications (H3K27me3) in chromatin, or by CO-IP to examine the interaction of RUNX1 and compressor complex, and by molecular method to verify the binding of RUNX1 to the regulatory DNA of STAT1 or IRF3 suppresses gene expression. Also, members of the RUNX family are often in contact with the STAT1 [66–68]. However, STAT1 was known to only act as an upstream effector of RUNX1 so far. For example, STAT1 regulates megakaryopoiesis by altering the expression of RUNX1 [69]. In the antiviral innate response, VSV infection significantly induced downregulation of miR-27a through the IFN/JAK/STAT1/RUNX1 signaling pathway in macrophages to inhibit type I IFN production [26]. Our study is the first report that the expression of STAT1 is regulated by RUNX1, which is likely related to the specific regulatory role of RUNX1 in lung epithelial cells. The mechanism of how IAV infection induces RUNX1 expression and how RUNX1 regulates the IFN signaling pathway is still elusive, which requires further investigation in the future. Besides, as a transcription factor, RUNX1 is functionally associated with the immune system development and critical for inducing the production of many immune genes, which suggests RUNX1 may also be involved in other signaling pathways during influenza infection. It is also an interesting direction in our future research.

## Conclusion

**Fig. 6:**
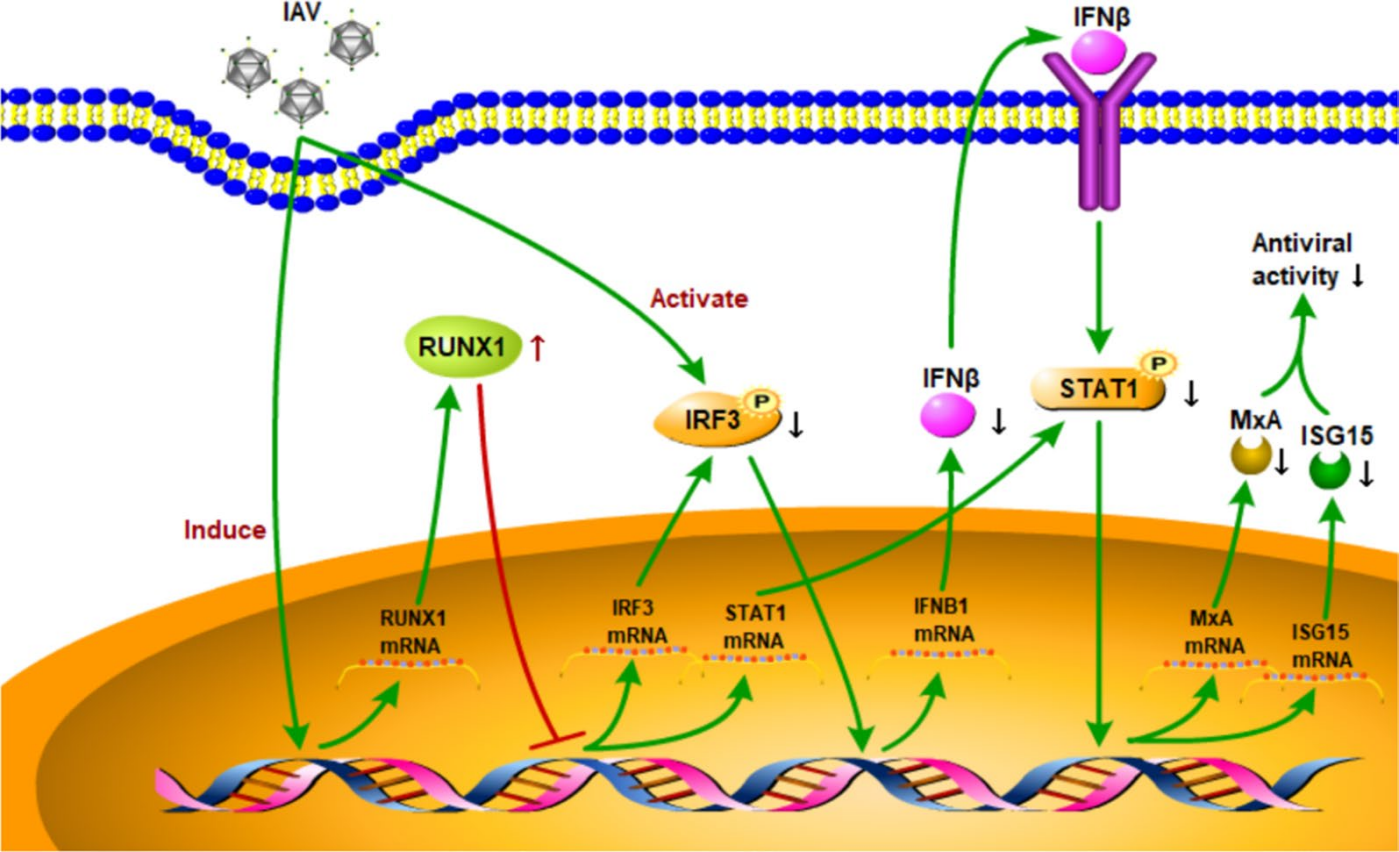
RUNX1 inhibits the antiviral immune response against IAV by attenuating type I interferon signaling. RUNX1 expression was induced by IAV infection. And then RUNX1 attenuates type I interferon signaling to impede the antiviral immune response

In summary, our study demonstrated that induction of RUNX1 expression by IAV infection helped IAV to escape host anti-viral response (Fig. 6). These findings provide a novel insight that RUNX1 may play a key regulatory role in innate immunity during virus infection.

## Data Availability

All data generated or analyzed during this study are included in this published article.

## Abbreviations

IAV: Influenza A virus
PR8: Influenza virus A/Puerto Rico/8/34 (H1N1)
ZJ163: Influenza A/Zhejiang/163/2020 (H3N2)
JSC1: Influenza A/swine/Jiangsu/C1/2008 (H9N2)
CA04: Influenza A/California/04/2009(H1N1)
MOI: Multiplicity of infection
IFN: Interferon
IFN-β: Interferon β
IRF3: Interferon regulatory factor 3
NP: Nucleocapsid protein
M1: Matrix protein 1
ISG: Interferon stimulated gene
